# Sex differences in molecular pathways underlying cardiovascular health in Black Americans

**DOI:** 10.21203/rs.3.rs-8391154/v1

**Published:** 2026-01-19

**Authors:** Harriet NA Blankson, Cecilia Delmer, Rashi Verma, Emine Guven, Kimberly Rooney, Andrea Pearson, Peter Baltrus, Arshed A Quyumi, Priscilla Pemu, I.King Jordan, Taylor Herman, Robert Meller, Charles D Searles

**Affiliations:** Morehouse School of Medicine; Morehouse School of Medicine; Morehouse School of Medicine; Morehouse School of Medicine; Emory University; Morehouse School of Medicine; Morehouse School of Medicine; Emory University; Morehouse School of Medicine; Georgia Institute of Technology; Morehouse School of Medicine; Morehouse School of Medicine; Emory University

**Keywords:** Sex Differences, Cardiovascular Health, Transcriptomics, Cytoskeletal, Immune Regulation, Black adults

## Abstract

**Background:**

Black Americans face a high burden of cardiovascular disease (CVD), with more than 60% of Black adult women affected. However, sex-specific molecular mechanisms underlying poor cardiovascular health (CVH) in this population remain largely unknown. In this study, we examined sex-specific transcriptomics signatures associated with CVH among Black adult men and women.

**Methods:**

Whole blood RNA-sequencing was performed on 373 Black adults. CVH was assessed using the American Heart Association Life’s Simple 7 (LS7) score. Differential gene expression (DGE) analysis comparing participants with poor-to-intermediate CVH (LS7 < 10) versus ideal CVH (LS7 scores ≥ 10) was conducted using LIMMA. Sex-stratified functional enrichment analysis was conducted using FGSEA and ClueGo. Shared differentially expressed genes (DEGs) were evaluated using fixed-effects meta-analysis. Upstream transcription factor, cytokine, and kinase activities were inferred using DoRothEA and OmniPath to assess sex-specific gene expression regulation at the transcriptional, and post-transcription level.

**Results:**

Among females, 430 DEGs were identified and indicated activation of RUNX2, PBX3, TFAP4 and enrichment of actin cytoskeletal pathways, consistent with vascular remodeling. In males with poor-to-intermediate CVH, 344 DEGs were detected and indicated inferred activation of GATA4, MAZ, and SOX10 and enrichment of pathways related to cardiac conduction and cellular metabolism. Thirteen DEGs were shared across sexes, including upregulation of *DNAJC6, KANK2, SPTB*, and MSTRG.22508, reflecting conserved stress response programs involving cytoskeletal remodeling and membrane stabilization. Although both sexes with poor-to-intermediate CVH exhibited suppression of adaptive immune effectors, in females the downregulation of *KIR2DL4, KLRF1*, and *SH2D1B* occurred alongside inhibition of RFX1/5, transcription factors essential for MHC class II expression and antigen presentation. In males, immune suppression was instead associated with inhibition of STAT1, indicating a shift away from cytokine-driven signaling.

**Conclusions:**

We identified distinct sex-specific molecular differences underlying CVH in a cohort of Black adults. Females with poor-to-intermediate CVH activate cytoskeletal and vascular remodeling pathways, consistent with structural reshaping. In contrast, males activate cardiac conduction and metabolic signaling programs, reflecting functional and bioenergetic compensation. Although both sexes exhibit immune repression in poor-to-intermediate CVH compared to ideal CVH, the mechanisms diverge, underscoring distinct sex-specific biological trajectories that may contribute to differential CVD risk and therapeutic effectiveness.

## Introduction

Cardiovascular disease (CVD) remains the leading cause of death worldwide, accounting for over 19 million deaths in 2021,(1–3) and presents significant health challenges across different demographic groups(4). In the United States, Black Americans are disproportionately affected, experiencing higher rates of hypertension, stroke, heart failure, and coronary artery disease than other demographic groups(4–6). The burden of CVD is especially pronounced among Black women, who exhibit higher cardiometabolic risk but also face poorer clinical outcomes compared to Black men and non-Black women(7, 8). Between 2000 and 2018, CVD mortality among Black women was two to three times higher than among white women(9, 10). Despite these differences in prevalence and prognosis, the underlying mechanisms, which are driven by a complex interplay of genetic, environmental, and socio-economic factors, remain incompletely understood.

High-throughput transcriptomic technologies provide power tools to investigate the molecular pathways underlying disease heterogeneity. RNA expression reflects the dynamic state of gene regulation influenced by both genetic background and environmental exposures, thereby integrating inherited and acquired risk factors(11, 12). Whole blood RNA sequencing (RNA-seq) enables comprehensive profiling of gene expression signatures associated with clinical phenotypes and therapeutic responses(13, 14). This approach has been informative in cardiovascular research, including heart failure(15) and coronary artery disease(16). Transcriptomic analyses have also revealed sex-specific differences in immune signaling, hormonal regulation and metabolic pathways(17, 18), underscoring relevance to precision medicine. Whole blood also captures circulating signaling molecules, including cytokines and miRNAs, that influence gene expression across tissues. (19) Since blood transcriptomic profiles often mirror tissue-specific patterns, these profiles may provide insights into broader physiological processes (20).

This whole blood transcriptome analysis expands on the Morehouse-Emory Cardiovascular (MECA) Center for Health Equity study, a community-based investigation of CVD risk and resilience to CVD among Black Americans(21). In the present study, cardiovascular health (CVH) was assessed using American Heart Association’s Life’s Simple 7 (AHA LS7) score, which incorporates seven modifiable lifestyle and clinical factors: smoking, physical activity, diet, body mass index (BMI), blood pressure, cholesterol levels, and blood glucose(22, 23). While LS7 provides a practical framework for clinical risk assessment and intervention, integrating it with transcriptomic profiling enables a unique opportunity to uncover the molecular mechanisms linking these risk factors to cardiovascular outcomes.

In this study, our objective was to identify differentially expressed genes and regulatory networks associated with CVH in Black adults, with a specific focus on identifying sex-specific transcriptomic profiles. By characterizing sex-specific molecular differences, our work provides a foundation for developing precision-targeted strategies to improve cardiovascular outcomes in Black adults.

## Methods

### Data and Sample collection

Participants of the MECA study completed study visits at either Emory University or Morehouse School of Medicine where they underwent a physical examination, blood draws, and standardized questionnaires. Vital signs and anthropometric measures were recorded. All blood samples were collected after > 6h of fasting, and fasting cholesterol and glucose levels were measured. Hypertension was defined as current use of anti-hypertensive medications, systolic blood pressure ≥ 130 mmHg, or diastolic blood pressure ≥ 80 mmHg. Diabetes mellitus was defined as current use of diabetes medications or fasting glucose ≥ 126 mg/dL. Hyperlipidemia was defined as current use of lipid-lowering medications or fasting total cholesterol ≥ 240 mg/dL. The study protocol was approved by the Institutional Review Boards at Morehouse School of Medicine (RB-FY2026–44) and Emory University (IRB00083584) and all participants provided written informed consent.

### Participant selection

The MECA study recruited adults ages 30 to 70 years who identified as Black and residents of the Atlanta metropolitan area for more than six years. The details of the study design have been previously described(21). Briefly, individuals with known CVD (e.g., myocardial infarction, congestive heart failure, cerebrovascular accident, coronary artery disease, peripheral arterial disease, atrial fibrillation, and cardiomyopathies), concomitant chronic illness (e.g., cancer, lupus, or HIV), substance abuse, psychiatric illness, pregnant or lactating females, and immobility such that physical activity could not be increased were excluded(21).

### Life’s Simple 7 metrics

**Figure 1:**
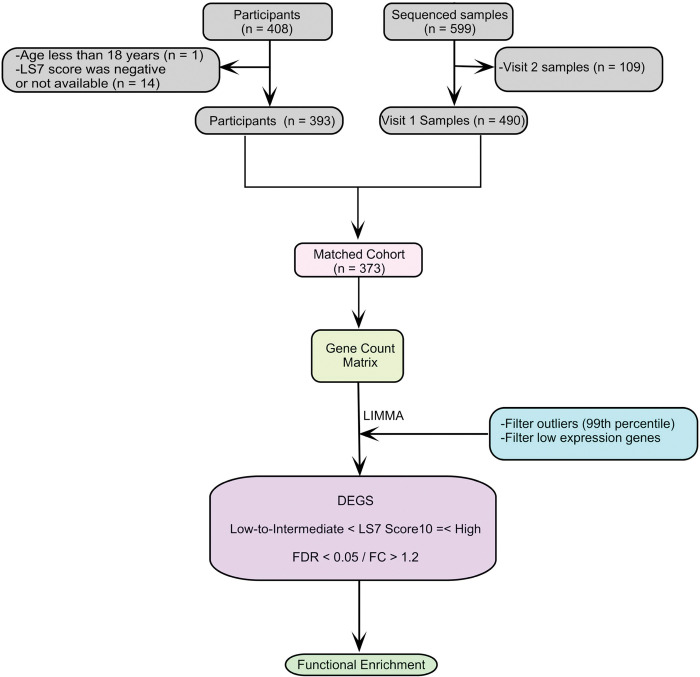
RNA-seq preprocessing and analysis pipeline. Overview of RNA-sequenced data processing, quality control, and differential expression workflow. CVH = cardiovascular health; LS7 = Life’s Simple 7, DEG = differentially expressed gene; FDR = false discovery rate; FC = fold change, n = number.

**Figure 2:**
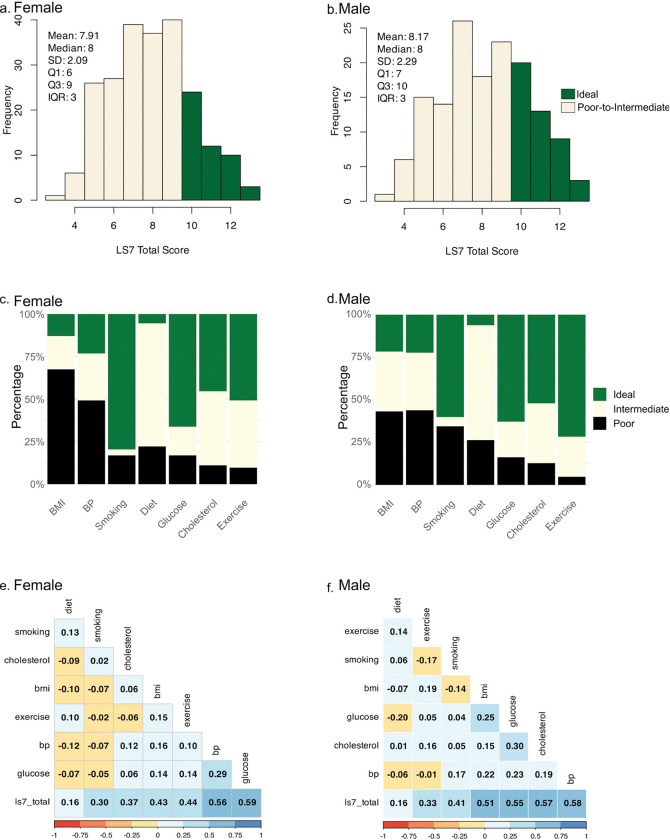
Distribution and Correlation of Life’s Simple 7 (LS7) Cardiovascular Health Metrics. Panel a and b show the distribution of LS7 total scores in females and male respectively. Panels c (females) and d (males) display the proportion of participants with ideal (green), intermediate (cream), and poor (black) LS7 subcomponent scores by sex. Panels e, and f illustrate Spearman correlation matrices of LS7 subcomponents for females, and males, respectively. Positive correlations are shown in blue and negative correlations in orange, with color intensity proportional to correlation strength. Together, these plots depict the overall CVH distribution and interrelationships among LS7 components by sex. BMI = body mass index; BP = blood pressure; CVH = cardiovascular health; LS7 = Life’s Simple 7.

The LS7 score, developed by the American Heart Association, is a validated metric for assessing CVH that incorporates both health behaviors (diet, exercise and smoking) and measurable health factors (BMI, cholesterol, fasting blood glucose and blood pressure)(24). Each LS7 component was scored as 0 (poor), 1 (intermediate) or 2 (ideal) based on established criteria,(21) and the total score was calculated by summing all seven subdomains, with a maximum score of 14 representing ideal CVH(22). None of the MECA study participants whose blood was studied had a LS7 score at the extremes (14 or < 3). Initially, the cohort was divided into tertiles based on natural breaks in total LS7 distribution: low (3–6), intermediate (7–9) and high LS7 scores (10–13). However, preliminary DGE analyses showed substantial overlap in the DEGs identified in males when comparing the low and intermediate versus high groups (Supplementary Fig. S1). Therefore, the low and intermediate LS7 groups were combined into a single category (poor-to-intermediate CVH, LS7 < 10) for comparison with the high LS7 group (ideal CVH, LS7 ≥ 10) (Figs. 1 and 2a). This data-driven approach minimized redundancy and strengthened group contrasts.

### RNA extraction

Blood was collected into PAXgene Blood RNA tubes (PreAnalytiX/Qiagen/BD Biosciences), and RNA was extracted using the PAXgene Blood RNA Kit (PreAnalytiX/Qiagen/BD Biosciences). RNA quality was assessed using a Fragment Analyzer (Agilent). One microgram of total RNA was subjected to ribosomal RNA (rRNA) and globin transcript depletion using the GLOBINclear Kit, human (ThermoFisher Scientific). Ten nanograms of the globin-depleted RNA was used as input for cDNA synthesis using the Clontech SMART-Seq v4 Ultra Low Input RNA kit (Takara Bio) according to the manufacturer’s instructions. Amplified cDNA was fragmented and appended with dual-indexed bar codes using the Nextera XT DNA library preparation kit (Illumina). Libraries were validated by capillary electrophoresis on a TapeStation 4200 (Agilent), pooled at equimolar concentrations, and sequenced with PE100 reads on an Illumina NovaSeq 6000, yielding ~ 30 million reads per sample on average.

### Data alignment and Differential Gene Expression Analysis

Raw RNA sequencing reads were initially pre-processed to remove rRNA that may still be present. Adapter sequences and low bases were trimmed using Trim Galore (0.6.4)(25). Cleaned reads were aligned to the human reference genome (Homo_sapiens.GRCh38.dna.primary_assembly.fa) using STAR (v2.7.3a)(26) and Bowtie2 (v2.3.5.1)(27), with the corresponding Ensembl annotation file (Homo_sapiens.GRCh38.109.gtf). Reads were aligned in two-pass using STAR(26). The aligned reads were then sorted, indexed, and filtered using SAMtools (v1.1.0)(28). Transcript assembly and quantitation were performed using StringTie (v2.2.1). Then prepDE.py was used to generate a unified count matrix for downstream analysis using R (v4.4.1). Fastq files were submitted to dbGAP (waiting on number).

Count-level RNA-seq data and phenotype data were analyzed using LIMMA in R (V4.4.1)(29). Samples were matched to the phenotype data, and duplicated samples were removed. Raw gene-level counts were imported into edgeR(30) using DGEList object. To remove extreme expression outliers, we calculated the maximum counts-per-million (CPM) value per gene and excluded genes above the 99th percentile. Low-expressed genes were filtered by retaining only those with CPM > 1 in at least the minimum group sample size. Counts were normalized using the trimmed mean of m-values (TMM) method to account for library size differences. The resulting filtered and normalized dataset was used as input for LIMMA-VOOM modeling. The design matrix included LS7 groups and age as covariates. Differential gene expression (DGE) analysis was performed separately for females and then males, using empirical Bayes moderation(31). Genes were considered significantly differentially expressed if at log_2_FC > 1.2 and FDR-adjusted P < 0.05 (Benjamini Hochberg correction(32)). Resulting differentially expressed genes (DEGs) lists were used in downstream analyses and visualization, including volcano plots. Data tables were used for subsequent analysis and plots.

### Fixed Effects Meta-Analysis of Differentially Expressed Genes Shared by Sexes

For the each shared DEG, the combined effect size across sexes was estimated using fixed-effects meta-analysis implemented in the metafor R package(33). The fixed-effects model was applied given the small number of groups (2 sexes) and low group heterogeneity. Genes with p < 0.05 and consistent directionality (same sign of log_2_FC across sexes) were interpreted as having sex-independent differential regulation. Forest plots were generated to visualize the direction and precision of effect estimates (Supplementary Fig. S2).

### Gene ontology and regulatory pathway analysis

Gene ontology (GO) and regulatory pathway analysis were conducted using fast gene set enrichment analysis (FGSEA)(34). Gene set used for enrichment was the GO terms (c5.all.v2023.2), KEGG (c2.cp.kegg_legacy.v2023.2) and Reactome (c3.all.v2023.2) from the Molecular Signatures Database(35). DGE analysis results were ranked by t-statistics. FGSEA(34) was applied to the entire dataset, utilizing 1000 permutations for gene sets to assess statistical significance(36). Pathways with adjust p-value ≤ 0.05 were considered significant, and top 10 upregulated and 10 downregulated pathways were visualized using dot plots.

To investigate the regulatory mechanisms underlying sex-specific transcriptional profiles, transcription factor (TF), cytokine, and kinase activity were inferred using decoupleR and DorothEA(37–39) framework and visualized in heatmap format. DEGs were used to build a comprehensive regulatory network with ClueGo(40) in Cytoscape (41).

### Statistical analyses

Statistical analyses (LIMMA, Spearman’s correlation, Student’s t-test, and Fisher’s exact test) were performed in R (version 4.4.1)

## Results

### Study Population Characteristics and CVH Assessment

Whole blood transcriptomic profiles were assessed in 373 self-identified Black adults living in the Atlanta metropolitan area. The mean age of participants was 53 years, and 60% (n = 225) were female (Table 1). Other demographic and clinical characteristics of the cohort have been described previously,(42) including prevalence of hypertension (53%), hyperlipidemia (31%), diabetes mellitus (21%), and current smoking (24%). The mean BMI was 33 kg/m^2^.

Total LS7 scores ranged from 3 to 13 in the cohort, with a median of 8 for both sexes (Fig. 2a-b). There was no significant difference in total LS7 scores between females and males (7.9 ± 2.1 vs 8.2 ± 2.3) (Table 1). However, females had higher average BMI and total cholesterol, while smoking prevalence was lower compared to males (Table 1, Fig. 2c-d).

Regarding LS7 subdomains, 67.6% (n = 152) of females and 43.2% (n = 64) of males had poor BMI (Fig. 2c-d). Poor blood pressure was also common, affecting 49.3% of females (n = 111) and 43.3% of males (n = 65). Ideal diet scores were rare in both sexes, 5.3% of females (n = 12) and 6.1% of males (n = 9). However, most participants had ideal fasting blood glucose (females: 66.2%, n = 149; males: 62.8%, n = 93) and ideal physical activity (females: 50.7%, n = 114; males: 71.6% n = 106). Ideal total cholesterol was achieved by 45% of females (n = 102) and 52% of males (n = 77).

Spearman correlation analysis revealed that fasting blood glucose and blood pressure were the strongest correlates of total LS7 scores in both sexes, while total cholesterol and BMI showed a stronger correlation with total LS7 score in males (Fig. 2e-f).

### Differential Gene Expression

**Figure 3:**
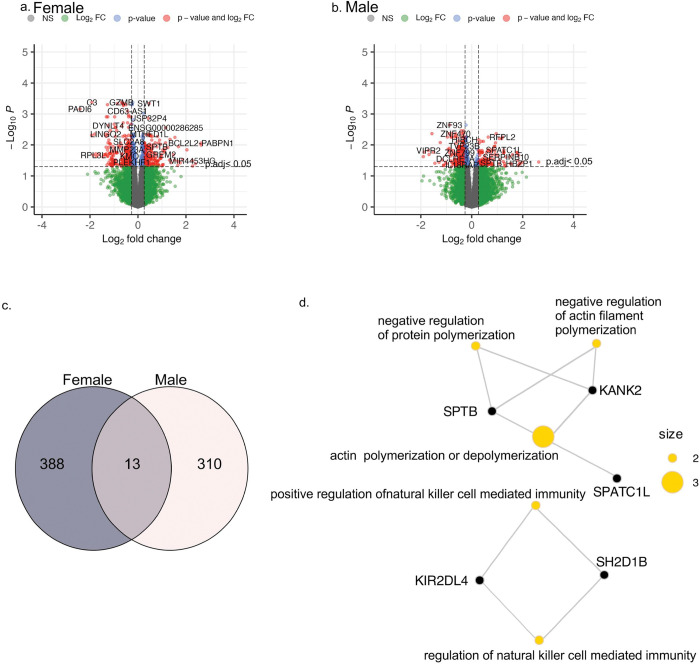
Sex-Stratified Differential Gene Expression and Functional Enrichment. Volcano plots illustrate differential gene expression between poor-to-intermediate and ideal cardiovascular health (CVH) in (a) females (b) and males. Genes with an adjusted p value < 0.05 were considered statistically significant; the horizontal dashed line marks the adjusted p value threshold (0.05). Red points represent significant differentially expressed genes (DEGs), whereas gray points denote nonsignificant genes. The Venn diagram (c) shows overlap of DEGs between females and males, identifying 13 shared genes. Network of Gene Ontology enrichment of these shared DEGs (d). CVH = cardiovascular health; DEG = differentially expressed gene; GO = Gene Ontology; LS7 = Life’s Simple 7.

The DGE analysis of low and intermediate (< 10 ) versus high (≥ 10) LS7 scores identified 430 DEGs in females and 344 DEGs in males (adj. P < 0.05, Fig. 3a-b, Supplementary Table 1&2). The number of upregulated DEGs in females (n = 180) was approximately 30% higher than in males (n = 130), while the number of downregulated DEGS in females (n = 250) was about 16% higher than in males (n = 214). Among the significant DEGs (adj. p < 0.05), fold change ranged from + 2.6 to −2.4 in females and + 2.6 to −1.9 in males. A greater proportion of DEGs in females were novel genes annotated only by StringTie (15%, n = 66) compared to males (12%, n = 42).

### Shared Differentially Expressed Genes

A total of 13 DEGs were shared between males and females with poor-to-intermediate CVH (LS7 scores < 10), of which *DNAJC6, KANK2, SPTB* and MSTRG.22508 were upregulated in both sexes, indicating conserved cellular stress response involving cytoskeletal remodeling, vascular development and membrane stabilization(43–45) (Fig. 3c-d, Table 2). A fixed effects meta-analysis confirmed consistent directionality for these genes (pooled log_2_FC ≈ 0.65–0.8, meta p < 0.001), indicating a sex-independent association with poor-to-intermediate CVH. Notably, *SPATC1L* displayed sex divergent regulation, upregulated (log_2_FC = 1.26, adj.P = 7.4E-05) in males, but downregulated in females (log2FC = −0.9, adj.P = 0.001), with heterogeneity observed in the meta-test, though not statistically significant (Supplementary Table S2&3, Table 2). All other shared DEGs (*ADGRA3, AKR1C3, B4GAT1, KIR2DL4, KLRF1, SH2D1B, WAPL-DT* and *ZNT595*) were consistently downregulated across sexes, reflecting coordinated suppression of immune-related and NK-cell activation pathways in poor-to-intermediate CVH individuals compared to ideal CVH (Fig. 3d).

### Functional enrichment analysis of Differentially Expression Genes – Gene Ontology Terms

When the gene expression profiles for females with poor-to-intermediate CVH were assessed for Gene Ontology (GO) enrichment analyses, we observed significant upregulation of GO terms related to cytoskeletal organization and actin filament dynamics, including negative regulation of actin filament polymerization (NES = 2.12, p.adj = 0.004), actin polymerization or depolymerization, exocytotic processes and hormone-responsive cytoskeletal remodeling (Table 3). In contrast, DEGs for males with poor-to-intermediate CVH showed upregulation of GO Terms such as cardiac-specific processes, such as regulation of heart rate by cardiac conduction (NES = 2.1, p.adj = 0.03), cardiac conduction and extracellular matrix organization (external encapsulating structure) (Table 4).

The highest downregulated GO terms in females with poor-to-intermediate CVH included immune and proteostasis pathways, particularly those involving immunoglobulin complex (NES = −2.36, p.adj = 2.04 × 10^− 6^), antigen processing and presentation of exogenous antigen and antigen binding (Table 3). In males with poor-to-intermediate CVH, the highest downregulated pathways were predominantly associated with mitochondrial and metabolic processes, such as translational initiation, tricarboxylic acid cycle, nucleoside phosphate metabolism, and reduced ER-Golgi trafficking (Table 4). Consistent with females, the immunoglobulin complex (NES = −2.24, p.adj = 3.5 × 10^− 5^) was downregulated in males indicating suppression of adaptive immune function in both sexes with poor-to-intermediate CVH.

**Figure 4:**
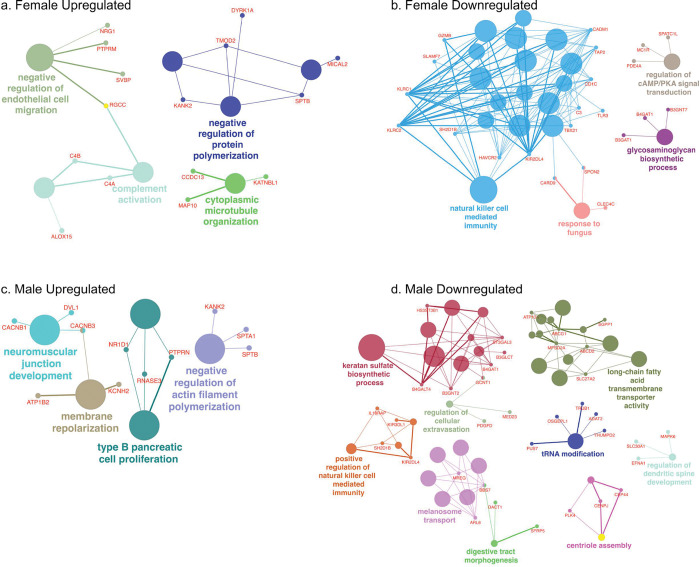
Sex-Specific Functional Enrichment Networks of Differentially Expressed Genes. ClueGO functional enrichment analysis showing biological processes enriched among up- and downregulated genes in females (a, b) and males (b, d) with poor-to-intermediate versus ideal cardiovascular health (CVH). Node size represents the number of genes within each pathway, and color denotes functional grouping or biological theme. CVH = cardiovascular health.

A ClueGO(40) network analysis, which informs how GO terms are linked, was performed to visualize functional interrelationships among up- and downregulated biological processes in males and females (Fig. 4). In females with poor-to-intermediate CVH, upregulation networks were mainly cytoskeletal and vascular remodeling, including negative regulation of endothelial cell migration, complement activation, negative regulation of protein polymerization and cytoplasmic microtubule organization, whereas males showed enrichment of neuromuscular junction development, negative regulation of actin filament polymerization and membrane repolarization pathways (Fig. 4a&c). Conversely, immune and extracellular biosynthetic processes were downregulated in females, while males exhibited suppression of metabolic and biosynthetic functions such as tRNA medication and long-chain fatty acid transport (Fig. 4b&d).

### KEGG Pathway Enrichment Reveal Sex-Specific Metabolic Signaling Differences

**Figure 5:**
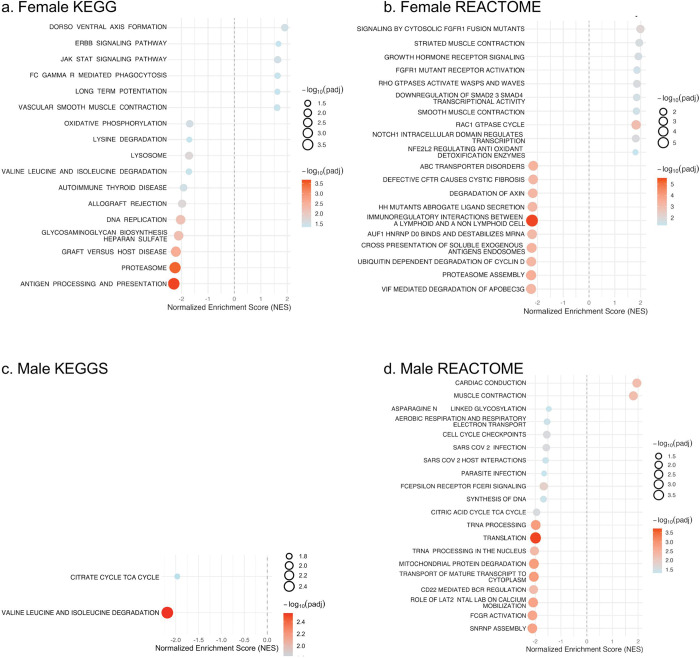
KEGG and Reactome Pathway Enrichment by Sex. Gene set enrichment analysis (GSEA) of KEGG and Reactome pathways in females (a–b) and males (c–d) with poor-to-intermediate versus ideal cardiovascular health (CVH). Node size indicates gene count per pathway; color scale reflects statistical significance (−log_10_ adjusted p value), and position reflects normalized enrichment score (NES). CVH = cardiovascular health; GSEA = gene set enrichment analysis; KEGG = Kyoto Encyclopedia of Genes and Genomes; NES = normalized enrichment score.

To extend the functional insights from GO analysis, KEGG pathway enrichment was performed to identify broader signaling and metabolic networks associated with poor-to-intermediate CVH in males and females. In females, upregulated pathways were predominantly linked to cellular signaling and vascular function, including dorsoventral axis formation, ERBB signaling, JAK-STAT signaling, long-term potentiation and vascular smooth muscle contraction (Fig. 5a). No KEGG pathways were significantly upregulated in males, suggesting that poor-to-intermediate CVH does not trigger strong coordinated increases in major biological pathways in men. On the other hand, downregulated KEGG pathways in females included antigen processing and presentation, proteosome, graft-versus-host disease, biosynthesis and DNA replication (Fig. 5a), reflecting suppression of immune, proteolytic and biosynthesis functions, in line with the GO terms analysis. Both males and females showed downregulation of valine, leucine and isoleucine degradation -branched-chain amino acids (BCAA) (Fig. 5a&c) (46). Although BCAAs support muscle protein synthesis and cellular energy, elevated circulating BCAA levels are associated with cardiometabolic dysfunction and increased CVD risk(46–54), suggesting impaired BCAA utilization in poor-to-intermediate CVH males and females. In males with poor-to-intermediate CVH, downregulated pathways were primarily metabolic, encompassing valines, leucine, and isoleucine degradation, and the citrate cycle, suggesting reduced mitochondrial energy metabolism in poor-to-intermediate CVH males also consistent with the GO terms analysis (Fig. 5c).

### Reactome Pathways Enrichment Highlights Distinct Signaling and Contractile Pathways by Sex

Reactome pathway analysis was performed to complement KEGG by showing in more detail how specific biological pathways are altered. Females with poor-to-intermediate CVH had upregulated Reactome pathways enriched for cytoskeletal and growth factor mediated signaling, including signaling by cytosolic FGFR1 fusion mutants, striated muscle contraction, growth hormone receptor signaling, RAC1 GTPase cycle, RHO GTPase activating WASPs and WAVES, which is consistent with observed GO enrichment for cytoskeletal organization (Fig. 5b). These pathways were consistent with the GO enrichment for cytoskeletal organization and actin filament dynamics and the KEGG enrichment for vascular and smooth muscle contraction, highlighting a coordinated activation of Rho-GTPase dependent cytoskeletal remodeling and hormone stress signaling in females with poor-to-intermediate CVH. Additional enrichment of striated muscle contraction and smooth muscle contraction indicates increased vascular and muscular contractile activity in females with poor-to-intermediate CVH. In males, upregulated Reactome pathways were limited to cardiac-specific contractile processes, specifically, cardiac contraction and muscle contraction, which was also consistent with the GO analysis cardiac conduction and extracellular matrix, suggesting increased myocardial electrical and mechanical signaling, which could be a male specific adaptive response to poor-to-intermediate CVH (Fig. 5d).

Downregulated Reactome pathways in females with poor-to-intermediate CVH were enriched for proteolytic and immune regulatory processes including VIF mediated degradation of APOBEC3G, proteosome assembly, immunoregulatory interactions between a lymphoid and a non-lymphoid cell (Fig. 5b). In males, downregulated Reactome pathways were primarily associated with mitochondrial, translational, and immune signaling functions, including SNRNP assembly, FCGR activation, translation, mitochondrial protein degradation, and role of LAT2 NTAL LAB on calcium mobilization (Fig. 5d).

### Upstream Regulator Analysis

To identify upstream molecular drivers of the observed transcriptional patterns, we performed regulator enrichment analyses, including transcription factor (TF), cytokine, and kinase activity inference, to determine key signaling regulators associated with poor-to-intermediate CVH in males and females.

**Figure 6:**
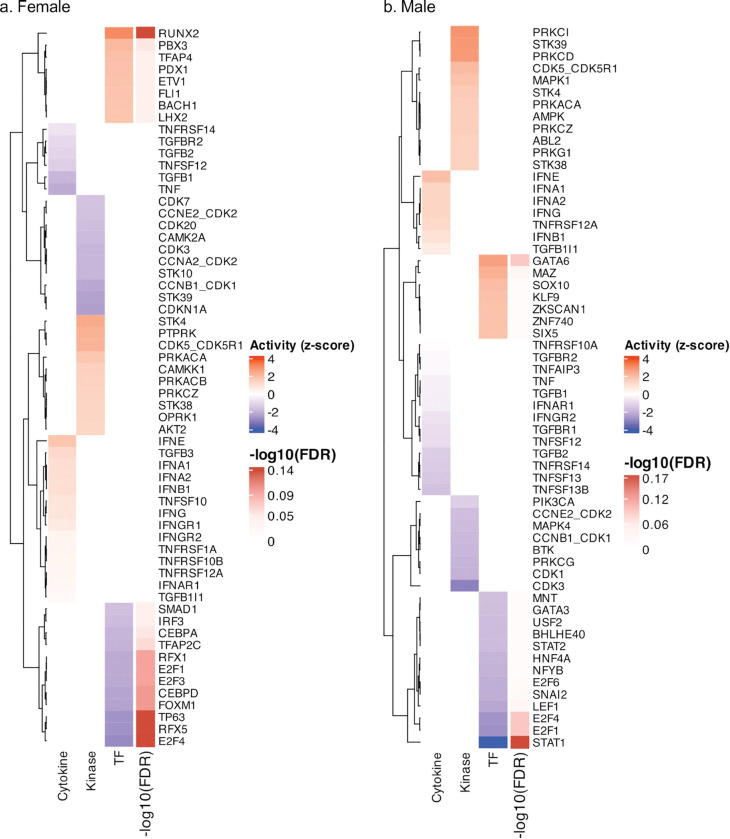
Sex-Specific Transcription Factor, Cytokine, and Kinase Activity Profiles. Heatmaps of inferred transcription factor (TF), cytokine, and kinase activity in females (a) and males (b) with poor-to-intermediate versus ideal cardiovascular health (CVH). Regulator activity was estimated using decoupleR with the DoRothEA and OmniPath knowledge bases. Red indicates activation and blue indicates inhibition (z-score scale). Right-side color bars indicate −log_10_(FDR) values from upstream regulator analysis, with darker shades denoting higher significance. CVH = cardiovascular health; TF = transcription factor.

In females, TF activity analysis inferred activation of RUNX2, PBX3, and TFAP4 (Fig. 6a), regulators that control cytoskeletal organization, vascular remodeling, and stress-responsive gene expression (55–57). On the other hand, CEBPA, CEBPD, E2F1–4, FOXM1, TFAP2C, TP63, and RFX1/5 were inhibited, which is consistent with the observed suppression of immune and cell-cycle pathways (58–62). Cytokine activity inference showed activation of TGFB3, IFNA1/2, IFNB1, IFNG, and TNFRSF10A/B/12A, while canonical inflammatory (TNF) and SMD-dependent TGF-β signaling components (TGFB1/2, TGFBR2) were inhibited(63–68), suggesting bias toward non-canonical, focal-adhesion linked remodeling. Correspondingly, kinase analysis identified activation of STK4/38, PTPRK, CDK5-CDK5R1, PRKACA/B, and CAMKK1, highlighting MAPK, AKT, and PKA/aPKC signaling cascades that likely drive vascular contractile and actin reorganization programs(69–71).

In males, TF activity analysis inferred activation of GATA6, MAZ, and SOX10, regulators associated with cardiac and vascular differentiation as well as neuromuscular conduction (72–77), while HNF4A, NFYB, E2F1/4/6, LEF1, SNAI2, and STAT1 were inhibited, indicating broad suppression of transcriptional programs governing metabolism, cell-cycle regulation, and immune signaling(78–81) (Fig. 6b). Cytokine analysis revealed limited activation of interferon and TNF receptor pathways (IFNG, IFNE, TNFRSF12A, TGFB1|1) but inhibition of TNF, TGFBR1, IFNAR1, and IFNGR2, reflecting reduced inflammatory tone(64, 65, 68, 81). Kinase activity in males was nominated by activation of MAPK1(ERK2), AMPK, PKA (PRKACA/B), aPKC (PRKCI/Z), PRKCD, and PRKC1, consistent with enhanced cardiac conduction and contractile signaling, whereas PIK3CA, MAPK4, BTK, and several CDKs were inhibited, indicating suppression of proliferative and immune signaling. While some of the regulators did not meet FDR thresholds, it is expected in enrichment-based inference. Also, the directional activity patterns were coherent with the observed sex-specific DEGs and pathway pathways enrichments.

## Discussion

In this study of 373 Black adults, we compared sex-specific whole blood transcriptomic profiles in participants with poor-to-intermediate CVH to those with ideal CVH. Although the distribution of total LS7 scores was similar between males and female participants, we identified distinct sex-specific gene expression patterns. Both males and females with poor-to-intermediate CVH had suppression of immune related and proteostatic pathways, but the upstream regulatory profiles and biological emphasis differed between sexes. Females had a structural and cytoskeletal remodeling phenotype, whereas males displayed a cardiac conduction and metabolic adaptation phenotype. These findings highlight sex-specific transcriptional, signaling, and kinase activities that may underlie different cardiovascular responses to lifestyle and cardiometabolic risk factors.

Males and female with poor-to-intermediate CVH had shared core cellular stress responses. This was reflected in the 13 DEGs common to both sexes. Two of these DEGs, *KANK2* and *SPTB* - involved in cytoskeletal remodeling, vascular development, and membrane stabilization were upregulated in both sexes(43, 44, 82). Another of the common DEGs, *SPATC1*, was upregulated in males and downregulated in females, which is appropriate given this gene’s role in spermatogenesis. Among the other genes downregulated in both sexes, *ADGRA3* has been shown to be involved in fat burning, and downregulation of this gene has been associated with obesity(83), an important risk factor of poor CVH. The downregulation of immune related genes (*SH2D1B* and *KIR2DL4*) links poor-to-intermediate CVH with reduced immunity in both males and females(84–87). This is also explicitly shown in high expression of immunoglobulin heavy and light chain genes in both males and females with high LS7 (Ideal CVH) scores and relatively decreased expression of these genes in males and females in lower LS7 scores (poor-to-intermediate CVH).

While we had expected to see transcriptomic profiles predominantly associated with inflammation, our data indicate predominance of transcriptomic profiles associated with structural adaptation in females with poor-to-intermediate CVH. Females showed activation of TFs (RUNX2, PBX3 and TFAP4) involved in osteogenic and vascular remodeling(55–57), accompanied by inhibition of TFs (E2F, CEBP, and RFX families) that coordinate immune and cell-cycle programs(58–62). Notably, suppression of RFX1 and RFX5 - key regulators of MHC class II expression - aligns with the decreased expression of antigen presentation and immunoglobulin complex genes, supporting relative immune suppression in females with poor-intermediate CVH(60–62). Males, in contrast, exhibited activation of GATA6, MAZ, and SOX10 - a regulatory network promoting cardiac contractility and neuromuscular signaling(72–77). In parallel, inhibition of STAT1 and HNF4A reflected suppression of metabolic and immune function(78–81). Interestingly, the transcriptomics profiles of males with poor-to-intermediate CVH, similar to that of females, indicated inhibition of E2F family of TFs, which have been shown to have a protective role in preventing cardiomyocyte hypertrophy(88).

At the post transcriptional level, that is changes that occur after mRNA is produced, both sexes displayed changes in immunoglobulin complex and antigen presentation pathways, but the regulation of these pathways appeared to differ. Females with poor-to-intermediate CVH had downregulation of *KIR2DL4, KLRF1*, and *SH2D1B* along suppression of RFX1/5 signaling, implying reduced activation of MHC class II and B-cell genes(86, 87, 89, 90). This shows post-transcriptional repression of adaptive immune effectors to maintain endothelial integrity during stress. In males, decreased expression of *B4GAT1* and *ADGRA3* and inhibition of STAT1 signaling suggested dampening of cytokine dependent transcription and interferon signaling(83, 91). Overall, immune gene repression appears to be conserved between males and females, but immune gene repression is mediated through different processing and transcriptional silencing in males versus post-transcriptional dampening via proteostatic control in females.

## Conclusion

In summary this work reveals that poor-to-intermediate CVH elicits sex-specific transcriptomic programs involving vascular remodeling, kinase and TF activity and cytokine networks. Females engage cytoskeletal and vascular remodeling programs and TGFB-RUNX2 signaling, while males emphasize cardiac conduction and metabolic compensatory pathways through ERK2-AMPK-GATA6 signaling, both coupled to suppress immune transcriptional activity. These differences highlight the potential importance of sex-specific precision medicine strategies, integrating molecular and behavioral data to tailor interventions that mitigate cardiovascular risk in diverse populations.

### Limitations

While this study provides valuable insights into sex-specific molecular correlates of CVH in Black adults, several limitations should be acknowledged. First, the FGSEA and enrichment analyses relies on existing gene annotations and pathway databases, which may not fully capture novel or context-specific interactions. Additionally, further validation of these pathways in independent cohorts is necessary to confirm their relevance. Future research should focus on integrating these findings with proteomic and metabolomic data.

Another limitation is the lack of longitudinal data, which prevents us from assessing how transcriptomic changes overtime correlate with the development or prevention of CVDs in individuals with high or poor-to-intermediate LS7 scores. Since this is a cross-sectional study, all clinical and transcriptomic measurements represent a single point in time, and we lack information about the duration or consistency of the participants’ lifestyles. Additionally, the lifestyle factors were self-reported and may not accurately reflect participants’ actual behaviors, potentially introducing reporting bias.

The absence of FDR-significant regulators likely reflects the conservative multiple-testing correction inherent to enrichment-based upstream analysis rather than a lack of true biological signal. Notably, the predicted activation patterns showed strong internal consistency with DEGs and pathway enrichments, supporting biological relevance of these regulatory networks.

### Clinical and Biological Relevance

Together, these findings indicate that poor-to-intermediate CVH in females is associated with a gene expression profile consistent with stress-driven vascular remodeling, whereas males engage contractile and metabolic compensatory pathways within cardiac tissue. In both sexes, these adaptations are coupled with suppressed immune transcriptional activity. These results suggest that sex differences arise not only from hormonal influences, but also from distinct transcriptional and signaling networks. Overall, poor-to-intermediate CVH is characterized by increased structural repair processes and reduced immunological activity.

## Supplementary Material

Supplementary Files

This is a list of supplementary files associated with this preprint. Click to download.

• supplementaryfig.pdf

• Supplementarytable.xls

## Figures and Tables

**Table 1:** Distribution and comparison of Clinical measures among study Participants BMI = body mass index, SBP = systolic blood pressure, DBP = diastolic blood pressure, HDL = high density lipoprotein, LDL = low density lipoprotein, LS7 = life simple 7, N = number, SD = standard deviation.

Total number of participants	148	225	
Age (years)	52.53 ± 10.51	53.62 ± 10.25	0.319
**Clinical Measures**			
BMI (kg/m^2^)	30.10 ± 6.59	34.60 ± 8.62	1.2x10^−7^*
Glucose (mg/dL)	102.82 ± 32.10	102.28 ± 39	0.888
SBP (mmHg)	132.26 ± 19.61	129.56 ± 19.47	0.192
DBP (mmHg)	79.68 ± 11.59	80.90 ± 11.77	0.326
Years Smoked (years)	9.28 ± 12.40	3.92 ± 9.07	2.29x10^−6^*
HDL (mg/dL)	55.78 ± 19.28	58.48 ± 15.60	0.137
LDL (mg/dL)	108.05 ± 38.44	117.48 ± 32.48	1.15x10^−2^*
Triglycerides (mg/dL)	107.30 ± 75.47	99.56 ± 45.58	0.218
Cholesterol (mg/dL)	185.46 ± 41.93	196.07 ± 37.75	1.15x10^−2^*
LS7 Total Score	8.17 ± 2.29	7.91 ± 2.09	0.255

P-values were calculated using the two-sample t-tests, and statically significant values were < 0.05 and marked *.

**Table 2:** Heterogeneity analysis of common differentially expressed genes in males and females logFC_= Log2 fold change se = standard error, p = p-value

Gene	logFC Female	logFC Male	Meta logFC	Meta SE	Meta p-value	Consistency
ADGRA3	−1.2638462	−1.2880897	−1.2744465	0.25128566	3.94E-07	TRUE
AKR1C3	−0.5784595	−0.4145407	−0.5060374	0.08262534	9.10E-10	TRUE
B4GAT1	−0.2846451	−0.3448068	−0.3108695	0.06914057	6.92E-06	TRUE
DNAJC6	0.59268818	0.73121454	0.65305189	0.14276674	4.78E-06	TRUE
KANK2	0.71891867	0.80813988	0.75785486	0.16620402	5.12E-06	TRUE
KIR2DL4	−1.1521196	−1.2369734	−1.189208	0.1933893	7.78E-10	TRUE
KLRF1	−0.3972859	−0.4642975	−0.4265114	0.09178079	3.37E-06	TRUE
MSTRG.22508	0.47361841	0.59287208	0.52556376	0.109439	1.57E-06	TRUE
SH2D1B	−0.4707316	−0.5202137	−0.4923344	0.08802647	2.23E-08	TRUE
SPATC1L	−0.8981771	1.26176526	0.04044289	0.20989822	0.85E-07	FALSE
SPTB	0.78834466	0.75932866	0.77564282	0.15112251	2.86E-07	TRUE
WAPL-DT	−0.5422633	−0.6436078	−0.5864529	0.1301706	6.63E-06	TRUE
ZNF595	−0.6536046	−0.5042423	−0.5877143	0.09389103	3.86E-10	TRUE

**Table 3:** Top 10 Upregulated and Downregulated Gene Ontology Terms in Females based on all genes pval = p-value, padj = p-adjusted value, ES = enrichment score, NES = normalized enrichment score. Table is ordered by normalized enrichment scores.

Pathway	pval	padj	ES	NES
NEGATIVE REGULATION OF ACTIN FILAMENT POLYMERIZATION	2.25E-05	0.004	0.53	2.12
NEGATIVE REGULATION OF SIGNAL TRANSDUCTION BY P53 CLASS MEDIATOR	3.81E-4	0.02	0.60	2.05
CELLULAR RESPONSE TO GROWTH HORMONE STIMULUS	1.38E-3	0.048	0.62	2
NEGATIVE REGULATION OF ACTIN FILAMENT DEPOLYMERIZATION	7.81E-4	0.03	0.52	1.93
EXOCYTIC PROCESS	1.88E-4	0.02	0.47	1.92
ACTIN POLYMERIZATION OR DEPOLYMERIZATION	4.23E-05	0.006	0.37	1.8
CELLULAR RESPONSE TO PEPTIDE HORMONE STIMULUS	1.08E-05	0.002	0.35	1.79
CYTOPLASMIC SIDE OF PLASMA MEMBRANE	1.53E-4	0.01	0.38	1.77
ACTIN FILAMENT	8.49E-4	0.03	0.41	1.76
REGULATION OF ACTIN FILAMENT LENGTH	3.83E-4	0.02	0.37	1.71
PROTEASOME REGULATORY PARTICLE	1.21E-4	0.01	−0.67	−2.07
DNA UNWINDING INVOLVED IN DNA REPLICATION	1.83E-4	0.02	−0.65	−2.08
REGULATION OF MITOTIC CELL CYCLE SPINDLE ASSEMBLY CHECKPOINT	6.75E-05	0.008	−0.68	−2.11
TRNA METABOLIC PROCESS	5.66E-09	1.01E-05	−0.42	−2.11
REGULATION OF NATURAL KILLER CELL MEDIATED IMMUNITY	3.18E-05	0.0047	−0.56	−2.12
ENDOPEPTIDASE COMPLEX	1.87E-06	6.0E-4	−0.50	−2.12
ANTIGEN BINDING	1.81E-07	1.6E-4	−0.46	−2.13
NATURAL KILLER CELL MEDIATED IMMUNITY	7.24E-06	0.002	0.53	−2.17
ANTIGEN PROCESSING AND PRESENTATION OF EXOGENOUS ANTIGEN	2.68E-05	0.004	−0.63	−2.23
IMMUNOGLOBULIN COMPLEX	5.70E-10	2.04E-06	−0.52	−2.36

**Table 4:** Top 10 Upregulated and Downregulated Gene Ontology Terms in Males based on all genes

Pathway	pval	padj	ES	NES
REGULATION OF HEART RATE BY CARDIAC CONDUCTION	2.16E-04	0.03	0.64	2.10
CARDIAC CONDUCTION	2.76E-04	0.03	0.47	1.91
EXTERNAL ENCAPSULATING STRUCTURE	3.68E-04	0.03	0.28	1.52
POST TRANSCRIPTIONAL REGULATION OF GENE EXPRESSION	1.49E-04	0.02	−0.29	−1.49
HP INTRAUTERINE GROWTH RETARDATION	1.23E-04	0.02	−0.28	−1.49
TRANSPORT VESICLE	5.52E-04	0.05	−0.30	−1.50
SMALL MOLECULE CATABOLIC PROCESS	5.35E-04	0.05	−0.30	−1.51
PURINE CONTAINING COMPOUND METABOLIC PROCESS	2.00E-04	0.02	−0.30	−1.52
HP AGE OF DEATH	1.34E-04	0.02	−0.30	−1.52
GOLGI VESICLE TRANSPORT	3.23E-04	0.03	−0.31	−1.52
COPII VESICLE COAT	6.25E-04	0.05	−0.67	−1.94
RNA METHYLATION	2.52E-05	0.01	−0.45	−1.95
COPII COATED VESICLE BUDDING	1.75E-04	0.02	−0.54	−1.96
NUCLEOSIDE MONOPHOSPHATE METABOLIC PROCESS	5.31E-05	0.01	−0.49	−1.97
NUCLEOSIDE DIPHOSPHATE METABOLIC PROCESS	1.15E-04	0.02	−0.58	−1.98
ER TO GOLGI TRANSPORT VESICLE MEMBRANE	7.28E-05	0.01	−0.53	−1.98
HP ACCESSORY ORAL FRENULUM	3.52E-04	0.03	−0.69	−1.98
TRICARBOXYLIC ACID CYCLE	8.38E-05	0.02	−0.62	−2.07
TRANSLATIONAL INITIATION	7.71E-07	6.89E-04	−0.47	−2.08
IMMUNOGLOBULIN COMPLEX	4.76E-09	3.40E-05	−0.50	−2.24

pval = p-value, padj = p-adjusted value, ES = enrichment score, NES = normalized enrichment score. Table is ordered by normalized enrichment scores.

## Data Availability

The datasets generated during the current study are under submission at dBgap. Other meta-data analyzed during the current study are available from the author on reasonable request.

## Abbreviations

AHA: American Heart Association
BCAA: Branched-chain amino acids
BMI: Body mass index
CPM: Counts-per-million
CVD: Cardiovascular disease
CVH: Cardiovascular health
DEGs: Differentially expressed genes
DGE: Differential gene expression
FC: Fold change
FGSEA: Fast gene set enrichment analysis
GO: Gene ontology
LS7: Life’s Simple 7
MECA: Morehouse-Emory Cardiovascular Center for Health Equity
NES: Normalized Enrichment Scores
TF: Transcription factor
TMM: Trimmed mean of m-values
